# Associations of childhood health and financial situation with quality of life after retirement – regional variation across Europe

**DOI:** 10.1371/journal.pone.0214383

**Published:** 2019-04-08

**Authors:** Claudia Börnhorst, Dörte Heger, Anne Mensen

**Affiliations:** 1 Leibniz Institute for Prevention Research and Epidemiology–BIPS, Bremen, Germany; 2 Leibniz Science Campus Ruhr and RWI–Leibniz Institute for Economic Research, Essen, Germany; 3 Ruhr-University Bochum, Bochum, Germany; Indiana University Purdue University at Indianapolis, UNITED STATES

## Abstract

Many studies have shown that childhood circumstances can have long term consequences that persist until old age. To better understand the transmission of early life circumstances, this paper analyses the effects of health and financial situation during childhood on quality of life after retirement as well as the mediating role of later life health, educational level, and income in this association. Moreover, this study is the first to compare these pathways across European regions. The analyses are based on data of 13,092 retirees aged ≥ 60 and ≤ 85 years from the fifth wave of the Survey of Health, Aging, and Retirement in Europe (SHARE) with full information on childhood and later life measures of health, educational level, financial situation, and quality of life as well as relevant covariates. Five European regions are studied: Central-Western Europe (Austria, Germany), Central-Eastern Europe (Czech Republic, Estonia, Slovenia), Northern Europe (Denmark, Sweden), Southern Europe (Italy, Spain), and Western Europe (Belgium, France, The Netherlands). Path analysis is used to identify the direct and indirect effects of childhood measures on quality of life. We find retirees’ quality of life to be associated with childhood finances and health in all five European regions. While both the direct and indirect effects of childhood health are rather moderate and homogeneous across regions, especially the direct effects of childhood finances on quality of life after retirement display a distinct North-South gradient being strongest in Southern Europe. Potential explanations for the regional variations are differences in the countries’ welfare systems.

## Introduction

It is well documented that those who are financially better off tend to be healthier [1] and more satisfied with their lives [2–4]. Moreover, various studies have shown that the financial situation does not only affect current but also future health and that a person’s socioeconomic status during childhood has a long-lasting impact on adult health [5, 6]. Similarly, childhood health is a significant predictor for later life health [7]. In general, Braveman and Barclay [8] affirm the need to employ life-course perspectives when considering health disparities. However, while the relationship between childhood circumstances and later life physical as well as mental health is well established [9–13], there are only very few studies looking at how childhood circumstances affect quality of life in older age [14, 15]. The present study aims at filling this gap by investigating the long-term effects of childhood health and financial situation on quality of life after retirement. Since quality of life is a comprehensive measure that includes aspects of control and self-realization, it is especially well-suited to assess the well-being of older adults [16]. Moreover, we examine whether these effects differ across European regions that vary in the comprehensiveness and generosity of their social systems.

Different conceptual models and hypotheses that link early life circumstances to later life outcomes exist in life-course epidemiology: the *critical/sensitive period model*, the *accumulation of risk model*, the *chains of risk model* and the *social drift model* [17]. The critical/sensitive period model proposes that exposures during certain time windows (e.g. childhood or adolescence) have solely an impact (“critical period”) or stronger impacts (“sensitive period”) on subsequent outcomes, e.g. health or quality of life in later life, compared to exposures during other time windows. Childhood can be considered a critical/sensitive period because the fundamentals in human and economic capital are built during this time [11]. Further, the adoption of health-related behaviours like eating habits are established [18]. Contrary, the accumulation of risk model argues that as the number, duration or severity of exposures increase over the life course, the cumulative damage increases additively, ultimately leading to poor later life outcomes. For instance, a long-lasting illness in early childhood or poor health behaviour in adolescence may both affect health in later life, but the exposures are not necessarily linked to each other. Going beyond a mere accumulation of risks, the chains of risk model (“pathway model”) refers to a sequence of linked exposures that increases disease risk. Social, biological or psychological chains of risk are possible and may contain mediating or modifying factors. For example, the socioeconomic background in childhood determines access to social and economic resources, which in turn affects the socioeconomic position in adolescence and adulthood and thereby later life health. The social drift model may be considered as a special case of the chains of risk model as it suggests early life health to indirectly affect later life health via acting on the later life socio-economic position [9, 19]. However, the mechanism linking childhood measures to later life outcomes are not yet fully understood.

The scarce evidence on the relationship between predictors of childhood socioeconomic status and quality of life in later life mostly points towards a positive association. Frijters, Johnston [20] demonstrate that e.g. the father’s social class at birth, health status at age seven or household income at age 16 can predict 7% of the variation in life satisfaction at ages 33 to 50. Layard, Clark [21] highlight emotional health to be the most important predictor of adult life satisfaction, while education plays a minor role concerning to their results. Using prospective data, Blane, Wahrendorf [22] found that social class during childhood and adulthood affects quality of life in early old age (at the age of 50) only modestly and mostly indirectly via contemporaneous factors like the financial situation and health status. Further, a recent study by Clark and Lee [23] emphasizes a positive association between parental income and parental education with the child’s well-being 50 years later. Recent studies by Wildman, Moffatt [14] and Kendig, Loh [15] analyse well-being in later life for the “baby boomer cohorts” in the UK and Australia, respectively. While both find evidence for the accumulation and the pathway model, the study by Wildman, Moffatt [14] finds also evidence for the critical period hypothesis. Wahrendorf and Blane [24] emphasize that disadvantaged circumstances during childhood cumulate during the life course. In particular, they found labour market disadvantage to partially mediate the relationship between childhood circumstances and quality of life in old age.

While these results demonstrate the importance of childhood circumstances on quality of life in mid and older age, previous studies were mainly based on standard regression frameworks, thereby failing to distinguish between direct and indirect effects. Building on the approach by Ploubidis, Benova [9], our study applies path analysis to investigate how health status and the financial situation during childhood (age 0 to 15) are associated with quality of life after retirement (age 60 to 85), considering educational level, post retirement net household income, and self-perceived health as potential mediators. We hence contribute to the literature by investigating quality of life in old age, while previous studies mostly consider the period of adulthood or early old age (e.g. Louis and Zhao [25]). Besides, we investigate the extent to which these associations differ between European regions representing different welfare systems and different economic circumstances during the respondents’ childhood and up to retirement. Since the welfare systems in Northern Europe put a large emphasize on redistribution and social security, we would expect childhood circumstances to have a relatively small impact on later life outcomes compared to the other regions. Further, due to the relatively low social expenditures in Southern European countries, lower levels of childhood finances or childhood health might be more likely to have long lasting consequences. To the best of our knowledge, such regional differences have not been studied before.

## Methods

### Data

The analysis was based on the data collected in the Survey of Health, Ageing and Retirement in Europe (SHARE) study (http://www.shareproject.org/), a multidisciplinary and cross-national panel database covering the population 50+ in Europe [26]. Depending on availability, national and municipal population registers or listings on dwellings are used as sampling frame to obtain probability samples with full population coverage. Country samples are drawn by random selection or multi-stage procedures. Detailed information is available in Malter and Börsch-Supan [27], Chapter 6. The SHARE study is subject to continuous ethics review. During Waves 1 to 4, SHARE was reviewed and approved by the Ethics Committee of the University of Mannheim. Wave 4 of SHARE and the continuation of the project were reviewed and approved by the Ethics Council of the Max Planck Society. For more details please see "overview and summary of the ethics approvals": http://www.share-project.org/organisation/dates-facts.html. The biennial survey waves include a wide variety of information on health, socio-economic status, and social family networks. Data are collected via computer-assisted personal interviews (CAPI) for the main interview and paper and pencil for a drop-off questionnaire. Wave 5 data collection took place in 2013 and included additional questions on early childhood conditions [27]. Participating countries were Austria, Belgium, Czech Republic, Denmark, Estonia, France, Germany, Israel, Italy, Luxembourg, Slovenia, Spain, Sweden, Switzerland, and The Netherlands. The SHARE data is available free of charge for scientific use after registration (information and questionnaires available at www.share-project.org).

### Study population

Data from SHARE wave 5 and easySHARE wave 5 are considered in the present analysis due to the collection of information on early childhood in that wave [28, 29]. The sample includes participants from 12 European countries. Israel was excluded as a non-European country; Luxembourg and Switzerland were not considered due to small sample size available for Luxembourg (n = 356) combined with a potential outlier problem since the Luxembourgian respondents tended to be more wealthy than those from other European SHARE countries and the Swiss health care system shows distinct features which made a classification impossible.

**Fig 1:**
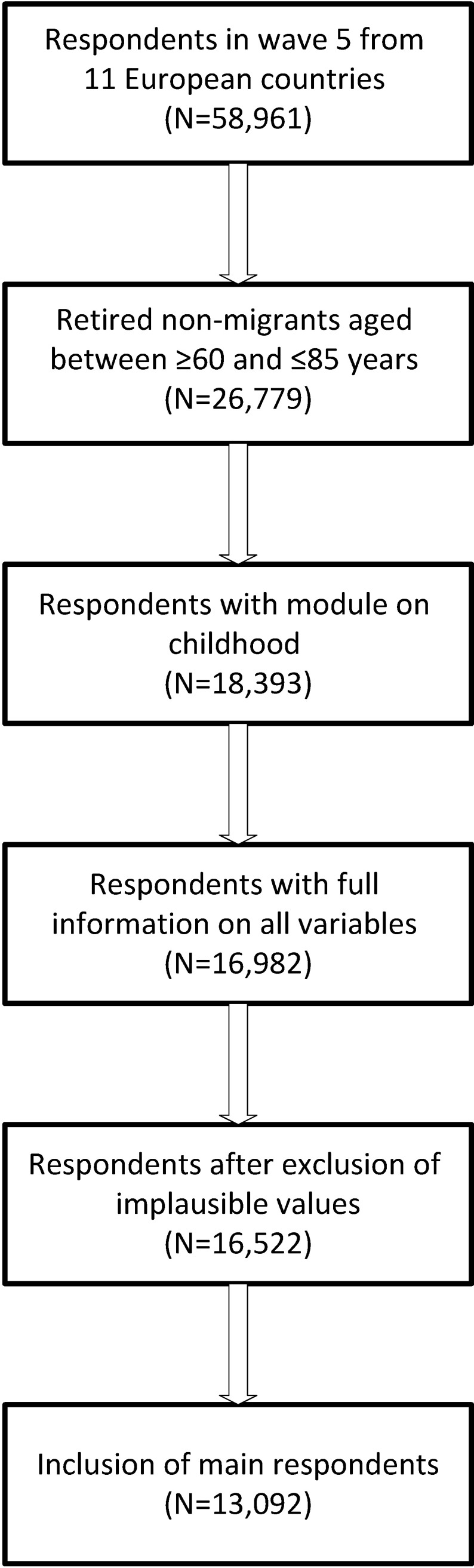
Flow chart representing the steps leading to the final study sample.

We restrict the study population to retired participants aged ≥ 60 to ≤ 85 in order to preclude (very) early retirement before the age of 60 and since persons older than 85 are a sparse group. Immigrants are excluded as their childhood circumstances are likely influenced by their country of birth rather than their country of residence, which would hamper an interpretation of the observed regional differences. Analyses restricted to migrants only were not feasible due to small sample sizes. Furthermore, only participants completing the module on childhood circumstances and with plausible information on the outcome variable, exposures, and covariates (see below) are considered. With the exception of the childhood measures, all variables refer to the respondents’ situation at the time of the interview. Only respondents with full information on the included variables are included. Since SHARE provides imputed income variables and missing data on non-monetary variables is low, the deletion of observations with missing information is unlikely to lead to a selected sample [30]. If multiple respondents out of the same household fulfilled these inclusion criteria, only the person being the main respondent is included. A flow chart displaying the steps leading to the final study sample of 13,092 respondents is given in Fig 1.

In order to assess regional differences, we group countries following the classification of the Esping-Andersen model of welfare states [31] that distinguishes between three types of welfare states–liberal, conservative-corporatist, and social-democratic. In conservative-corporatist welfare systems, e.g. the “Bismarck” countries, the state intervenes whenever families’ capacity to provide social protection is exhausted. Redistribution of incomes is modest. Contrary, social-democratic welfare systems found in the Scandinavian countries offer generous and universal benefits, which leads to a large redistribution of incomes. Within the group of the conservative-corporatist countries, Southern European countries are often seen as a distinct sub-group due to their lower level of social expenditures as a percentage of GDP [32, 33]. Similarly, Fenger [34] argues that the welfare states of post-communist Central and Eastern Europe countries represent a separate group since the level of social programs and the social situation are lower than in e.g. Western European countries. We therefore perform separate analyses for Northern Europe (represented in our sample by Denmark and Sweden), Southern Europe (Italy and Spain), and Central-Eastern Europe (Czech Republic, Estonia, and Slovenia). In addition, we divide the conservative-corporatist countries into a group of Western Europe (Belgium, France, and the Netherlands) and Central-Western Europe (Austria and Germany) to account for additional differences in culture and language based on regional proximity.

### Outcome variable

#### Quality of life

The CASP-12 score is used to measure quality of life. The measure was specifically developed for SHARE as a shortened version of the extensively studied and validated CASP-19 (for details, see Hyde, Wiggins [35]) to assess the quality of life of older adults. The measure is based on 12 questions on four aspects of quality of life (each assessed with three questions) **C**ontrol, **A**utonomy, **S**elf-Realization, and **P**leasure. The CASP-12 score is highly correlated with both the CASP-19 score and the Life Satisfaction Index [36, 37]. Questions include, e.g. whether the respondent thinks that age prevents him/her from doing things he/she likes to do (control), he/she can do the things he/she wants to do (autonomy), he/she looks forward to each day (pleasure) or whether he/she feels full of energy these days (self-realization). The full set of questions is listed in S1 Table. In each case, the answering categories are often, sometimes, rarely or never (coded from 1 to 4, with 4 indicating the most positive outcome). The CASP-12 score is the sum of respondent’s score on all 12 questions and ranges from 12 to 48 with higher scores indicating better quality of life [35, 38].

### Exposures

#### Childhood health

Respondents were asked to rate their general health from birth to age 15 as ‘excellent’, ‘very good’, ‘good’, ‘fair’ or ‘poor’ (coded as 5 = excellent to 1 = poor).

#### Childhood finances

Respondents were asked to rate their financial situation from birth to age 15 as ‘pretty well off financially’, ‘about average’ to ‘poor’ (coded as 3 = pretty well off financially to 1 = poor).

#### Self-perceived health

Analogue to the question on childhood health status, respondents rated their current health status as ‘excellent’, ‘very good’, ‘good’, ‘fair’ or ‘poor’ (coded as 5 = excellent to 1 = poor).

#### Household net income

Respondents reported their current income after taxes and social contributions capturing the notion of take-home pay. Yearly net household income is harmonized across European countries by adjusting for differences in spending power. It is further divided by the square root of the number of persons in the household to correct for household size. Extreme values are excluded to obtain a more homogenous sample and because outliers lead to unstable models estimates (country-specific 1^st^ and 99^th^ percentiles are used as cut-offs; in addition, yearly net incomes (corrected for household size) below 1,000 Euro and above 200,000 Euro are excluded). Details on the derivation of the income variables in SHARE and harmonization is given in Bertoni, Bonfatti [39]. Due to a relatively high number of missing values for monetary variables, the imputed income measures provided by SHARE are used (see De Luca, Celidoni [30] for details).

#### Education

Respondents’ highest educational level achieved is measured by the International Standard Classification of Education (ISCED) 1997 [40]. The variable is a generated variable provided in SHARE using country-specific information on the highest school leaving certificate, school degree or vocational training. The coding ranges from no education (0), to primary education (1), lower secondary education (2), (upper) secondary education (3), post-secondary education (4), first stage or tertiary education (5), and second stage of tertiary education (6). The categories “other” and “still in school” were set to missing.

### Covariates

Following previous literature (e.g. Ploubidis, Benova [9] and Pakpahan, Hoffmann [11]), age in years, sex, country of origin, a dummy indicating whether the respondent lives with a spouse/partner in the same household, the number of children still alive (including natural children, fostered, adopted and stepchildren), and a verbal fluency score as a measure of cognitive function are considered as covariates in the analysis. To assess verbal fluency, respondents were asked to enumerate animals resulting in a test score ranging from 0 to 100.

### Statistical analyses

**Fig 2:**
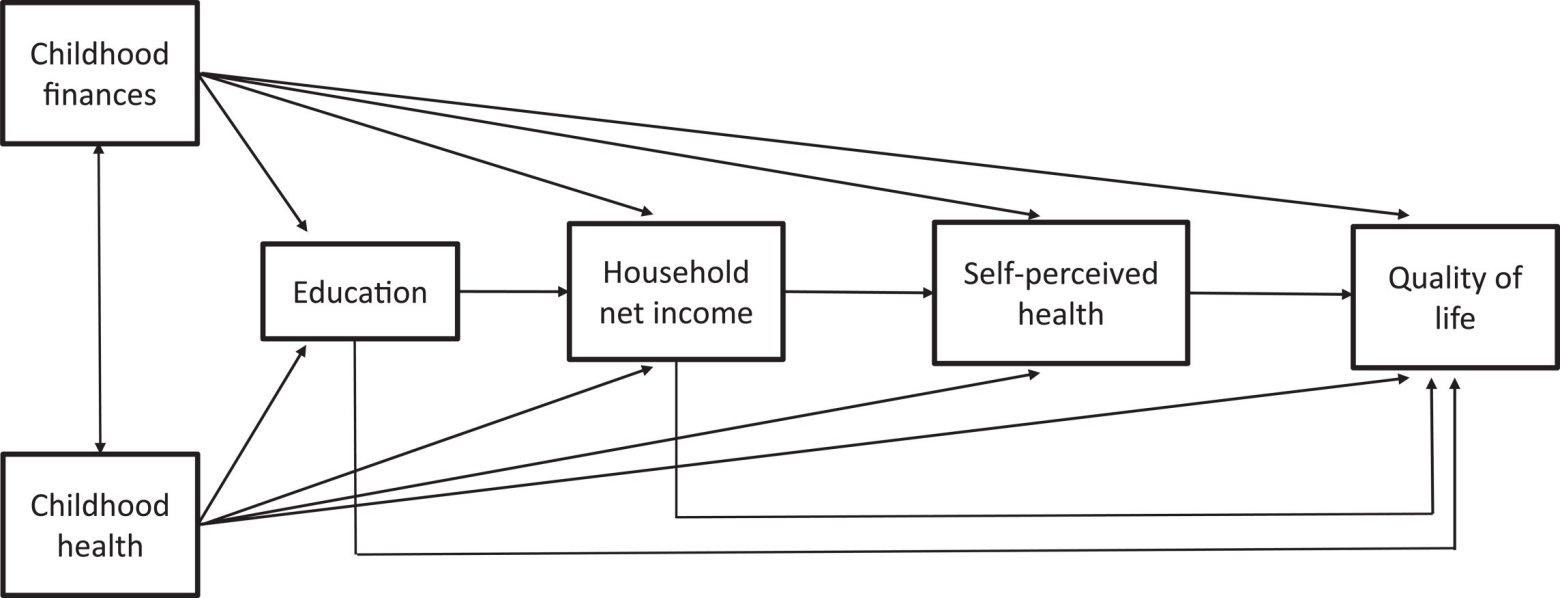
Theoretical model displaying the assumed associations of childhood financial situation and health with educational level, later net income, self-perceived health, and quality of life.

We use path analysis to test a theory-driven model linking childhood circumstances to quality of life after retirement. Path analysis has the advantage over linear regression that path coefficients are estimated via simultaneous equation estimation [41]. These models further allow the estimation of total, direct, and indirect effects. Our theoretical model is displayed in Fig 2. The focus lies on the association between childhood finances as well as childhood health on quality of life. As potential mediators, we consider educational level, household income and self-perceived health in later life. Childhood financial situation is assumed to affect quality of life after retirement directly but also via educational level, household income and self-perceived health. Likewise, childhood health is assumed to directly and indirectly affect quality of life via educational level, household income and self-perceived health. Childhood health and childhood finances are assumed to be correlated as the association may be bidirectional.

All variables considered are standardized across the entire sample before running the path model to allow for comparability of effect estimates among European regions and among predictor variables. An effect estimate hence refers to the effect obtained by one standard deviation (SD) increase in the variable of interest (SD calculated using data of all countries). Subsequently, path models are estimated stratified for the five European regions considered. Within these regions, inter-country differences are accounted for by inclusion of country dummies as adjustment terms. All models are adjusted for the covariates described above (see S2 Table for a more detailed description of the covariates being related to the different endogenous (outcome) variables in the model). S3 Table provides tables displaying variances/covariances among all variables considered in the path models for the five European regions. After running the a priori defined path model, some model modifications summarized in S2 and S4 Tables are applied based on both theoretical considerations and model fit indices. This includes changes in the covariates affecting the four endogenous variables quality of life, health, and net household income after retirement as well as educational level. In addition, certain (predicted) covariances are set to 0 to reduce model complexity and increase model fit. For parameter estimation, we use the Asymptotically Distribution Free (ADF) Estimator that relaxes the assumption of normally distributed outcome measures (self-reported health and educational level are not normally distributed). A Bentler-Bonett’s Normed Fit Index (NFI) > 0.99, Tucker-Lewis Index (NNFI) > 0.97, Comparative Fit Index (CFI) ≥ 0.99 and Root Mean Square Error of Approximation (RMSEA) ≤ 0.02 indicated a good model fit in all models estimated [42].

Mediation is assessed by computing estimates of direct, indirect, and total effects of the associations specified in the model. Direct effects represent associations between variables unmediated by any other variable in the model. Indirect effects represent mediated effects (or combined mediated effects for paths through multiple mediators). Total effects are the sum of the direct and indirect effects. It should be noted that use of the terms “direct effect” and “indirect effect” for describing effect estimates is standard terminology in path analysis. However, this does not necessarily imply causality of associations. For all models, path coefficients and corresponding 95% confidence intervals are reported. All analyses are performed using SAS statistical software version 9.4 (SAS Institute, Inc., Cary, NC). All path models are run using SAS Proc CALIS.

## Results

### Descriptive results

Descriptive statistics are shown in Table 1. Our sample consists of slightly fewer men (N = 6,244; 48%) than women (N = 6,848; 52%) though the sex distribution differs somewhat across regions. In Central-Eastern Europe, women are overrepresented (62%), while in Southern Europe men are overrepresented (62%). The average age is similar across regions ranging from 70.6 in Central-Western to 71.9 years in Southern Europe. Our main outcome, the quality of life index, is lowest in Southern and Central-Eastern Europe (average ratings of 35.2 and 36.0, respectively) and highest in Northern Europe (40.5). Study participants in Northern and Western Europe rate both their financial situation as well as their health during childhood higher than their counterparts from Southern and Central-Eastern Europe. The same holds for health after retirement: Respondents in Central-Eastern Europe rate their health poorer than respondents in the other European regions, while respondents in Northern Europe rate their health highest. Further, respondents in Western Europe have the highest household income, while it is far the lowest in Central-Eastern Europe. The educational level is lowest in Southern Europe (mean ISCED level: 1.5) and relatively similar in the other European regions (ranging from 2.8 in Western Europe to 3.3 in Central-Western Europe).

**Table 1:** Descriptive statistics.

	Northern EU (N = 1,662)		Central-Eastern EU (N = 4,387)		Southern EU (N = 1,980)		Western EU (N = 2,537)		Central-Western EU (N = 2,526)		All (N = 13092)	
**Male sex of the respondent (N, %)**	804	48.4	1645	37.5	1236	62.4	1309	51.6	1250	49.5	6244	47.7
**Age at interview** (mean, SD)	71.7	5.9	70.9	6.7	71.9	6.5	70.8	6.6	70.6	6.3	71.1	6.5
**Quality of life** (mean, SD)	40.5	4.9	36.0	6.2	35.2	6.0	38.6	6.0	39.9	5.6	37.7	6.2
**Childhood finances** (N, %)												
Poor	260	15.6	1699	38.7	814	41.1	574	22.6	779	30.8	4126	31.5
About average	1138	68.5	2412	55.0	1053	53.2	1494	58.9	1487	58.9	7584	57.9
Pretty well	264	15.9	276	6.3	113	5.7	469	18.5	260	10.3	1382	10.6
**Childhood finances** (mean SD)	2.0	0.6	1.7	0.6	1.6	0.6	2.0	0.6	1.8	0.6	1.8	0.6
**Childhood health** (N, %)												
Poor	35	2.1	231	5.3	41	2.1	65	2.6	81	3.2	453	3.5
Fair	117	7.0	584	13.3	95	4.8	177	7.0	289	11.4	1262	9.6
Good	267	16.1	1359	31.0	686	34.6	747	29.4	719	28.5	3778	28.9
Very good	368	22.1	1261	28.7	676	34.1	667	26.3	841	33.3	3813	29.1
Excellent	875	52.6	952	21.7	482	24.3	881	34.7	596	23.6	3786	28.9
**Childhood health** (mean, SD)	4.2	1.1	3.5	1.1	3.7	0.9	3.8	1.1	3.6	1.1	3.7	1.1
**Self-perceived health** (N, %)												
Poor	74	4.5	716	16.3	252	12.7	178	7.0	204	8.1	1424	10.9
Fair	328	19.7	1757	40.1	619	31.3	692	27.3	740	29.3	4136	31.6
Good	504	30.3	1457	33.2	772	39.0	1049	41.3	951	37.6	4733	36.2
Very good	447	26.9	348	7.9	255	12.9	422	16.6	476	18.8	1948	14.9
Excellent	309	18.6	109	2.5	82	4.1	196	7.7	155	6.1	851	6.5
**Self-perceived health** (mean, SD)	3.4	1.1	2.4	0.9	2.6	1.0	2.9	1.0	2.9	1.0	2.7	1.0
**Household net income** (mean, SD)	25025.1	12812.5	10890.2	6835.0	14033.8	8012	25546.0	20428.4	20549.5	10271.1	17863.7	13610.0
**Education according to ISCED-97** (mean, SD)	3.0	1.5	2.9	1.2	1.5	1.4	2.8	1.6	3.3	1.2	2.8	1.5
**Living with spouse/partner** (N, %)	1210	72.8	2599	59.2	1466	74.0	1644	64.8	1670	66.1	8589	65.6
**Verbal fluency score** (mean, SD)	22.2	6.5	21.5	6.8	15.2	6.2	19.4	6.3	22.5	7.2	20.4	7.1
**Number of children** (mean, SD)	2.3	1.2	2.1	1.1	2.1	1.4	2.2	1.4	2.0	1.4	2.1	1.3

### Path analysis

**Fig 3:**
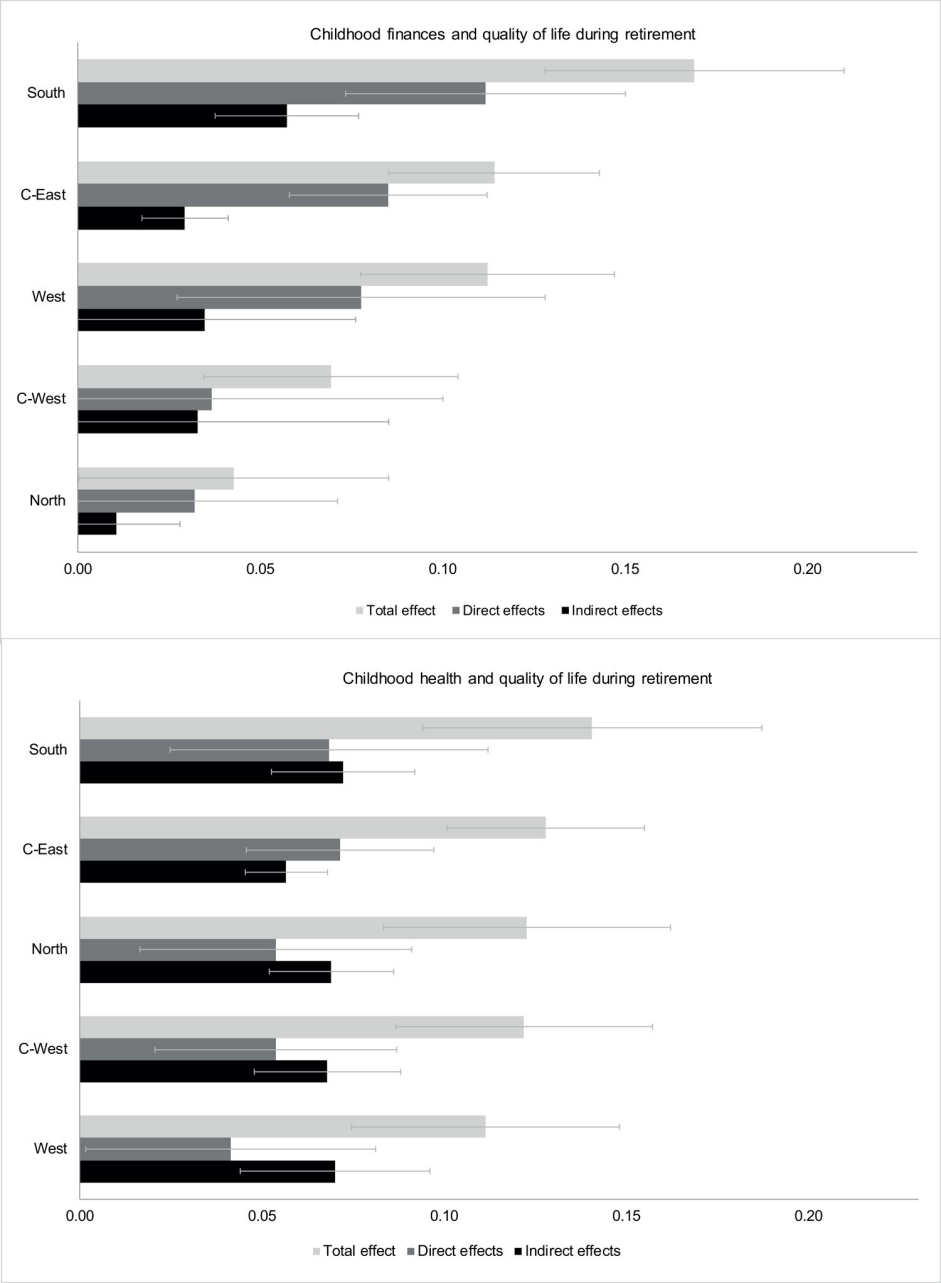
Effects of childhood circumstances on quality of life.

Next, we turn to the results of our path model. Our main results of interest are the effects of childhood finances and health on quality of life after retirement, which are presented graphically in Fig 3. In addition, Table 2 displays the results of the path analysis stratified by European region in more detail. Direct, indirect as well as total effects describing the associations of childhood finances, childhood health, educational level, net income, and health with quality of life after retirement are shown.

**Table 2:** Direct and indirect effects across European regions.

	Northern EU (N = 1,662)		Central-Eastern EU (N = 4,387)		Southern EU (N = 1,980)		Western EU (N = 2,537)		Central-Western EU (N = 2,526)	
	β	95% CI	β	95% CI	β	95% CI	β	95% CI	β	95% CI
**Direct effects**										
Child Finances ➔ Education	**0.16**	(0.108,0.213)	**0.10**	(0.079,0.127)	**0.24**	(0.195,0.282)	**0.14**	(0.104,0.176)	**0.11**	(0.084,0.146)
Child Finances ➔Net Income	**0.03**	(-0.014,0.070)	**0.00**	(-0.014,0.013)	**0.07**	(0.009,0.124)	**0.03**	(-0.023,0.086)	**0.03**	(0.002,0.057)
Child Finances ➔ Later Health	**0.03**	(-0.027,0.084)	**0.04**	(0.017,0.068)	**0.08**	(0.041,0.127)	**0.07**	(0.036,0.105)	**0.05**	(0.015,0.093)
Child Finances ➔ Quality of Life	**0.03**	(-0.007,0.071)	**0.08**	(0.058,0.112)	**0.11**	(0.074,0.150)	**0.08**	(0.027,0.128)	**0.04**	(-0.027,0.100)
Child Health ➔ Education	**0.04**	(-0.008,0.090)	**0.00**	(-0.027,0.019)	**-0.02**	(-0.060,0.029)	**0.08**	(0.037,0.114)	**0.03**	(-0.004,0.058)
Child Health ➔ Net Income	**0.05**	(0.018,0.088)	**0.00**	(-0.017,0.009)	**-0.02**	(-0.053,0.008)	**0.05**	(-0.003,0.112)	**0.03**	(0.007,0.061)
Child Health ➔ Later Health	**0.22**	(0.169,0.271)	**0.14**	(0.121,0.169)	**0.20**	(0.159,0.249)	**0.17**	(0.131,0.204)	**0.17**	(0.130,0.208)
Child Health ➔ Quality of Life	**0.05**	(0.017,0.091)	**0.07**	(0.046,0.097)	**0.07**	(0.025,0.112)	**0.04**	(0.002,0.081)	**0.05**	(0.020,0.087)
Education ➔ Net income	**0.19**	(0.153,0.233)	**0.12**	(0.106,0.140)	**0.06**	(-0.161,0.275)	**0.25**	(0.192,0.308)	**0.28**	(0.245,0.314)
Education ➔ Later Health	**0.09**	(0.040,0.145)	**0.11**	(0.043,0.185)	**0.04**	(-0.004,0.088)	**0.08**	(0.041,0.116)	**0.11**	(-0.038,0.251)
Education ➔ Quality of Life	**-0.05**	(-0.087,-0.014)	**0.05**	(0.017,0.084)	**0.04**	(-0.000,0.077)	**0.00**	(-0.286,0.282)	**-0.02**	(-0.455,0.425)
Net Income ➔Later Health	**0.14**	(0.073,0.207)	**0.16**	(-0.357,0.676)	**0.15**	(0.079,0.225)	**0.03**	(0.003,0.052)	**0.17**	(-0.308,0.652)
Net Income ➔ Quality of Life	**0.06**	(0.018,0.108)	**0.12**	(0.067,0.182)	**0.10**	(0.039,0.161)	**0.06**	(0.033,0.079)	**0.12**	(0.075,0.167)
**Indirect effects**										
Child Finances ➔ Education ➔Net Income	**0.03**	(0.019,0.043)	**0.01**	(0.009,0.016)	**0.01**	(-0.038,0.065)	**0.04**	(0.023,0.047)	**0.03**	(0.023,0.042)
Child Finances ➔ Education ➔ Net Income ➔ Later Health	**0.02**	(0.011,0.035)	**0.01**	(0.009,0.019)	**0.02**	(0.010,0.034)	**0.01**	(0.007,0.019)	**0.02**	(0.007,0.039)
Child Finances ➔ Educ ➔ Net Income ➔ Later Health ➔ QoL	**0.01**	(-0.007,0.028)	**0.03**	(0.018,0.041)	**0.06**	(0.038,0.077)	**0.03**	(-0.007,0.076)	**0.03**	(-0.020,0.085)
Child Health ➔ Education ➔ Net Income	**0.01**	(-0.002,0.018)	**0.00**	(-0.003,0.002)	**0.00**	(-0.005,0.003)	**0.02**	(0.008,0.030)	**0.01**	(-0.001,0.016)
Child Health ➔ Education ➔ Net Income ➔ Later Health	**0.01**	(0.004,0.021)	**0.00**	(-0.006,0.003)	**0.00**	(-0.010,0.001)	**0.01**	(0.003,0.013)	**0.01**	(-0.008,0.028)
Child Health ➔Educ ➔ Net Income ➔ Later Health ➔ QoL	**0.07**	(0.052,0.086)	**0.06**	(0.046,0.068)	**0.07**	(0.052,0.092)	**0.07**	(0.044,0.096)	**0.07**	(0.048,0.088)
Education ➔ Net Income ➔ Later Health	**0.03**	(0.013,0.041)	**0.02**	(-0.044,0.083)	**0.01**	(-0.024,0.042)	**0.01**	(0.001,0.013)	**0.05**	(-0.086,0.182)
Education ➔ Net Income ➔ Later Health ➔ QoL	**0.05**	(0.029,0.065)	**0.07**	(0.054,0.084)	**0.02**	(-0.013,0.063)	**0.05**	(0.031,0.062)	**0.09**	(0.067,0.110)
Net Income ➔ Later Health ➔ QoL	**0.04**	(0.020,0.061)	**0.06**	(-0.143,0.271)	**0.06**	(0.029,0.086)	**0.01**	(0.001,0.020)	**0.06**	(-0.109,0.231)
**Total effects**										
Child Finances ➔Quality of Life	**0.04**	(0.000,0.085)	**0.11**	(0.086,0.143)	**0.17**	(0.128,0.210)	**0.11**	(0.078,0.147)	**0.07**	(0.035,0.104)
Child Health ➔ Quality of Life	**0.12**	(0.083,0.162)	**0.13**	(0.101,0.155)	**0.14**	(0.094,0.187)	**0.11**	(0.075,0.148)	**0.12**	(0.087,0.157)
Education ➔ Quality of Life	**0.00**	(-0.042,0.035)	**0.12**	(0.084,0.155)	**0.06**	(0.010,0.117)	**0.04**	(-0.240,0.328)	**0.07**	(-0.367,0.514)
Net Income ➔ Quality of Life	**0.10**	(0.056,0.151)	**0.19**	(-0.027,0.403)	**0.16**	(0.088,0.227)	**0.07**	(0.042,0.091)	**0.18**	(0.007,0.358)
Later Health ➔ Quality of Life	**0.29**	(0.254,0.326)	**0.40**	(0.370,0.432)	**0.38**	(0.337,0.420)	**0.38**	(0.341,0.413)	**0.35**	(0.319,0.387)
**Model fit indices**										
RMSEA and 90% CI	0.01 (0; 0.037)		0.02 (0; 0.038)		0.02 (0; 0.033)		0.02 (0; 0.038)		0 (0; 0.033)	
Bentler-Bonett NFI	1.00		1.00		0.99		1.00		1.00	
Tucker-Lewis Index (NNFI)	0.99		0.99		0.98		0.98		1.00	
Bentler CFI	1.00		1.00		1.00		1.00		1.00	

#### Childhood financial situation

The upper part of Fig 3 shows the total, direct, and indirect effects of childhood finances on quality of life for the five European regions. Our results indicate a positive direct effect on quality of life after retirement in Southern, Central-Eastern, and Western Europe. An increase in childhood finances by 1 SD has a direct effect of 0.11, 0.08, and 0.08 SD, respectively, on quality of life corresponding to an increase in the CASP-12 score by between 0.5 and 0.7 units. In contrast, the direct effects are much smaller in Northern (β = 0.03; 95% confidence interval [-0.01;0.07]) and Central-Western (β = 0.04; 95% CI [-0.03;0.10]) Europe. The indirect effects of childhood finances on quality of life are smaller compared to the corresponding direct effects in all European regions. By far the largest indirect effect can again be found in Southern Europe (β = 0.06; 95% CI [0.04;0.08]) and the smallest one in Northern Europe (β = 0.01; 95% CI [(-0.01;0.03]), while being of similar size in Central-Eastern, Central-Western and Western Europe (β = 0.03). Consequently, the total effect (sum of direct and indirect effects) of childhood finances on quality of life after retirement also displays a strong North-South gradient.

#### Childhood health

The effect of childhood health on quality of life after retirement is shown in the lower part of Fig 3. In general, the effects are more homogeneous and of similar size across the European regions. The direct effects of a 1 SD increase in childhood health range from 0.07 SD in Central-Eastern and Southern Europe to 0.04 SD in Western Europe corresponding to an increase in the CASP-12 score by 0.4 and 0.2 units, respectively. The indirect effects of childhood health on quality of life amount up to 0.07 SD in all regions except Central-Eastern Europe (β = 0.06; 95% CI [0.05;0.07]). The total effects of a 1 SD increase in childhood health on quality of life range from 0.14 SD (0.9 units on original scale) in Southern Europe to 0.11 SD (0.7 units) in Western Europe.

#### Mediators

Childhood finances show a positive direct effect on education in all European regions. The effect is largest in Southern Europe, where a 1 SD increase in childhood finances is associated with an increase of 0.24 SD in educational level, corresponding to an increase in ISCED-97 by 0.4 original units. In contrast, the direct effect of childhood health on education is close to zero except for Western Europe (β = 0.08; 95% CI [0.04;0.11]).

Furthermore, we observe large direct effects of a 1 SD increase in educational level on net income, especially in Central-Western, Western and Northern Europe (β = 0.28, 0.25 and 0.19, meaning that an increase in ISCED-97 categories by 1.5 leads to an increase in net income by 3811, 3403 and 2586 Euro, respectively). In contrast, the direct effect is smaller in Southern Europe (β = 0.06; 95% CI [-0.16;0.28]).

In addition, we find a positive direct effect of net income on quality of life in all European regions, ranging from 0.06 SD in Northern and Western Europe to 0.12 SD in Central-Eastern and Central-Western Europe. Further, large and positive direct effects of self-perceived health on quality of life are observed in all European regions.

Lastly, an indirect effect of net income on quality of life via later health is only observed in Southern Europe (β = 0.06; 95% CI [0.03,0.09]), while the largest indirect effect of educational level on quality of life via net income and later health are observed in Central-Western and Central-Eastern Europe (β = 0.09 and 0.07, respectively).

## Discussion and conclusion

In this paper, we present evidence that quality of life after retirement is associated with one’s financial situation and health during childhood in all European regions studied. In general, we find support for the critical/sensitive period, the accumulation model as well as the chains of risk model as discussed in detail below, thereby being in line with the emerging view that these models are rather complementary than incompatible [15]. However, the magnitude of the effects and therefore the strengths of the different pathways vary across European regions. While we find relatively homogeneous effects for childhood health on quality of life, the direct and total effects of childhood finances on quality of life after retirement show a clear North-South gradient. In particular, childhood finances have a comparatively large direct effect on quality of life after retirement in Southern, Central-Eastern and Western Europe, which is in line with the critical/sensitive period hypothesis. In contrast, only weak effects are observed in Northern Europe. Conde-Sala, Portellano-Ortiz [43] interpret the CASP-12 score as indicating low quality of life for a score < 35, moderate for a score ≥ 35 to < 37, high for a score ≥ 37 to < 39, and very high for a score ≥ 39. The observed increase in the CASP-12 score in Southern Europe by 0.7 units would hence indicate an upward movement of approximately one third of a category. Though this increase might be modest, it still represents a remarkable influence of childhood circumstances even considering that any intermediate variables not considered in the present analysis are implicitly included in the direct effect. Increasing efforts to fight childhood poverty may hence have long-run consequences on quality of life even after retirement, especially in Southern and Central-Eastern Europe.

Moreover, a pathway model showing that childhood finances influence quality of life after retirement not only directly but also via later life factors is mostly supported in Southern Europe, while we do find smaller and relatively homogenous effects for the remaining European regions. Further, the similar indirect effects of childhood health on quality of life in all European regions point to pervasive evidence for the social drift hypothesis, i.e. the negative effect of poor health during childhood is transmitted via other outcomes, which finally leads to lower quality of life.

Present health is generally seen as a central aspect of quality of life [22], a finding that is also confirmed by our analysis. In contrast, education and income after retirement arguably also affect quality of life indirectly by providing financial security, enabling the purchase of goods and services that improve quality of life or a better understanding of how to benefit from the available resources. In most regions, we find both income after retirement and educational level positively affect quality of life, though the effects are much smaller compared to self-perceived health. Although our model does not provide information on other possible factors contributing to quality of life beyond the included mediators, participation in society and daily activities are aspects of quality of life that go beyond physical or mental health or purchasable goods and services [44]. Whether inclusion and participation are possible, likely depends on a country’s institutional setting. Income, education, and health may become less important predictors of later quality of life in more inclusive societies. Similarly, generous welfare systems are often viewed to promote an equitable society. The idea is that the institutional setting creates a level playing field, where every individual has the same chance to succeed in life. In this optimal scenario, later life outcomes should not be predetermined by socioeconomic characteristics of one’s parents or by childhood circumstances. With respect to childhood finances, our predefined hypothesis is confirmed. The discovered North-South gradient with respect to childhood finances on quality of life may be linked to the traditionally generous social systems in Northern relative to the Southern European countries that may compensate for a more strained financial situation during childhood [32, 45]. However, with respect to childhood health, even a comprehensive and high quality health care system may not be able to completely alleviate poor (permanent) health outcomes during childhood or compensate for poor health behaviour [46, 47].

Comparison with previous studies is hampered by the use of different outcome measures (see, e.g., [9–11]), i.e. most previous investigations focussed on associations between early life circumstances and later life (mental) health, physical activity or morbidity. For instance, Huisman, Kunst [48] show that differences in socioeconomic inequalities in morbidity among older adults exist across European countries. Besides, previous studies used different data sets for investigating associations in different countries [49], were unable to analyse the effects on a disaggregated level due to problems of statistical power [11] or used linear regression frameworks instead of path analysis [50]. An exception of the latter is Hoffmann, Kröger [51] who analysed the relative importance of social causation (the effect of socioeconomic status on health) and health selection (the effect of health on socioeconomic status) for three European regions. Their results show no clear differences between the studied regions. However, how these results translate to later life quality of life remains unknown. The two studies looking at quality of life as an outcome use data from Australian baby boomers [15] and an industrial city in north-east England [14, 15]. In line with our results, both studies find childhood circumstances to be associated with older age quality of life and that this relationship works through different pathways.

While we consider the use of the path analysis model as one of the strengths of the paper, several limitations remain. For instance, it should be noted that strong a priori assumptions about relationships among variables are made when applying path models. However, data-driven alternatives often fail to identify plausible pathways and our theoretical model was constructed based on previous literature providing ample evidence for the assumed relationships. Further, it is important to note that the direct effects summarize all effects of a variable on a certain outcome not mediated through intermediate variables considered in the model. Due to data limitations as well as limits to the model’s flexibility, we were unable to test the exact pathways of how childhood circumstances affect quality of life after retirement but can only infer potential pathways via the included mediators. Hence, it might be that there is no “real” direct link between the childhood conditions and later life outcomes but another indirect link through further intermediate variables not considered in the present analysis such as, for instance, lifestyles, periods of unemployment or disability, divorce or the availability of social resources. Such potentially unobserved and omitted variables are arguably highly correlated with education, income or health. Including all three variables as mediators thus helps to reduce the impact of omitted variables on our direct effects estimates since the mediators partly serve as proxy for the omitted variables. Similarly, unobserved differences in individuals’ preferences might influence individuals’ behaviour such as, e. g., the reason for retirement, which is not available for the full sample and therefore could not be included in the analysis. By restricting the sample to retirees aged 60 to 85, we created a more homogeneous sample, which reduces potential selection problems. Besides, including self-perceived health as mediator explicitly accounts for differences in health status. In addition, while the availability of childhood measures is rare and represents a strength of this study, we cannot rule out measurement error or recall bias. Hence, it should also be acknowledged that both measures are retrospective reports and measurement errors may be correlated due to common method variance. Lastly, instead of comparing European regions, a cross-country comparison would have been interesting. However, due to the smaller sample sizes estimations for single countries led to unstable model estimates in some countries, which corroborated the decision to focus on European regions. Furthermore, the intended comparison of different welfare systems motivated our classification of countries. Also, when estimating the models stratified by sex the model fit was unsatisfactory such that no sex-specific results can be presented.

Despite some limitations, our study extends previous findings on how childhood circumstances track into later life. Using path analysis, we are able to identify the direct and indirect effects of childhood finances and childhood health on quality of life. With respect to the direct effects of childhood finances on quality of life after retirement, we observe a distinct North-South gradient, which may be linked to differences in the countries’ welfare systems. Our outcome of interest, quality of life after retirement, goes beyond other commonly used measures such as physical or mental health. The detected differences in the associations of the financial situation during childhood with quality of life across European regions highlight the need for further research to explore the underlying reasons of these differences and the need for early-life interventions which implicitly increase later life quality of life.

## Supporting information

S1 Table List of relevant CASP variables in wave 5 of SHARE. (DOCX)

S2 Table Exposures and covariates selected a priori for the three endogenous variables as well as after model modification based on fit indices and theoretical considerations (final models). (DOCX)

S3 Table Variances/covariances among variables included in the path models. (XLSX)

S4 Table Covariances set to zero in a priori defined model as well as after model modification based on fit indices and theoretical considerations (final models). (DOCX)
