# Supplementary material for: Associations of childhood health and financial situation with quality of life after retirement – regional variation across Europe
Source: PLoS One. 2019 Apr 8;14(4):e0214383. doi: 10.1371/journal.pone.0214383 (PMC6453524; doi:10.1371/journal.pone.0214383)
Supplement: S1 Table — (DOCX) [file pone.0214383.s001.docx]

S1 Table. List of relevant CASP variables in wave 5 of SHARE

| **Question** | **Question text** | **Subscale** |
| --- | --- | --- |
| AC014 | How often do you think your age prevents you from doing the things you would like to do? | Control |
| AC015 | How often do you feel that what happens to you is out of your control? | Control |
| AC016 | How often do you feel left out of things? | Control |
| AC017 | How often do you think that you can do the things that you want to do? | Autonomy |
| AC018 | How often do you think that family responsibilities prevent you from doing what you want to do? | Autonomy |
| AC019 | How often do you think that shortage of money stops you from doing the things you want to do? | Autonomy |
| AC020 | How often do you look forward to each day? | Pleasure |
| AC021 | How often do you feel that your life has meaning? | Pleasure |
| AC022 | How often, on balance, do you look back on your life with a sense of happiness? | Pleasure |
| AC023 | How often do you feel full of energy these days? | Self-Realization |
| AC024 | How often do you feel that life is full of opportunities? | Self-Realization |
| AC025 | How often do you feel that the future looks good for you? | Self-Realization |

Source: Mehrbrodt T, Gruber S, Wagner S. Scales and Multi-Item Indiactors: Share Manuals 2017. Available from: http://www.share-project.org/fileadmin/pdf_documentation/SHARE_Scales_and_Multi-Item_Indicators.pdf.
