# Supplementary material for: Associations of childhood health and financial situation with quality of life after retirement – regional variation across Europe
Source: PLoS One. 2019 Apr 8;14(4):e0214383. doi: 10.1371/journal.pone.0214383 (PMC6453524; doi:10.1371/journal.pone.0214383)
Supplement: S2 Table — (DOCX) [file pone.0214383.s002.docx]

S2 Table. Exposures and covariates selected a priori for the three endogenous variables as well as after model modification based on fit indices and theoretical considerations (final models).

| **Endogenous variable** | **Exposures and covariates in a priori defined path model** | **Exposures and covariates in the final path model (Northern Europe)** | **Exposures and covariates in the final path model (Western Europe)** | **Exposures and covariates in the final path model (Southern Europe)** | **Exposures and covariates in the final path model (Central-Eastern Europe)** | **Exposures and covariates in a priori defined path model (Central-Western Europe)** |
| --- | --- | --- | --- | --- | --- | --- |
| Quality of life | Later life health, net household income, childhood finances, childhood health, educational level, sex, country dummies, living with spouse, number of children, age, fluency score | Later life health, net household income, childhood finances, childhood health, educational level, sex, country dummies, living with spouse, number of children, age, fluency score | Later life health, net household income, childhood finances, childhood health, educational level, sex, country dummies, living with spouse, number of children, fluency score | Later life health, net household income, childhood finances, childhood health, educational level, sex, country dummies, number of children, age, fluency score | Later life health, net household income, childhood finances, childhood health, educational level, sex, country dummies, living with spouse, number of children, age, fluency score | Later life health, net household income, childhood finances, childhood health, educational level, sex, living with spouse, number of children, age, fluency score |
| Later life health | Net household income, childhood finances, childhood health, educational level, sex, country dummies, living with spouse, number of children, age, fluency score | Net household income, childhood finances, childhood health, educational level, sex, country dummies, living with spouse, number of children, age, fluency score | Net household income, childhood finances, childhood health, educational level, sex, country dummies, living with spouse, number of children, age, fluency score | Net household income, childhood finances, childhood health, educational level, country dummies, number of children, age, fluency score | Net household income, childhood finances, childhood health, educational level, sex, Estonia dummy, living with spouse, number of children, age, fluency score | Net household income, childhood finances, childhood health, educational level, sex, country dummies, living with spouse, age, fluency score |
| Net household income | Child finances, childhood health, educational level, sex, country dummies, living with spouse, number of children, age, fluency score | Child finances, childhood health, educational level, sex, country dummies, living with spouse, number of children, age, fluency score | Child finances, childhood health, educational level, sex, country dummies, living with spouse, number of children, age, fluency score | Child finances, childhood health, educational level, sex, living with spouse, number of children, age, fluency score | Child finances, childhood health, educational level, sex, country dummies, living with spouse, number of children, age, fluency score | Child finances, childhood health, educational level, sex, living with spouse, number of children, age, fluency score |
| Educational level | Childhood finances, childhood health, fluency score, number of children, sex, country dummies | Childhood finances, fluency score, number of children, sex, country dummies, living with spouse, | Childhood finances, fluency score, number of children, sex, country dummies, living with spouse, | Childhood finances, fluency score, number of children, sex, country dummies | Childhood finances, fluency score, number of children, sex, country dummies, living with spouse, | Childhood finances, fluency score, number of children, sex, country dummies |

Country dummies relate to the different regions, e.g. for North Europe consisting of Denmark and Sweden in our data a country dummy for Sweden was added to the model; analogously dummies were added for the other regions
