# Supplementary material for: Associations of childhood health and financial situation with quality of life after retirement – regional variation across Europe
Source: PLoS One. 2019 Apr 8;14(4):e0214383. doi: 10.1371/journal.pone.0214383 (PMC6453524; doi:10.1371/journal.pone.0214383)
Supplement: S4 Table — (DOCX) [file pone.0214383.s004.docx]

**S4 Table. Covariances set to zero in a priori defined model as well as after model modification based on fit indices and theoretical considerations (final models).**

| **Covariances set to zero in a priori defined model** | **Covariances set to zero in model for Northern Europe** | **Covariances set to zero in model for Central-Eastern Europe** | **Covariances set to zero in model for Southern Europe** | **Covariances set to zero in model for Western Europe** | **Covariances set to zero in model for Central-Western Europe** |
| --- | --- | --- | --- | --- | --- |
| Country vs. age, country vs. sex, sex vs. living with spouse, sex vs. number of children, age vs. number of children, fluency score vs. number of children | Age vs. number of children, Sweden vs. sex, living with spouse vs. childhood finances, age vs. childhood finances, sex vs. number of children, fluency score vs. number of children, Sweden vs. living with spouse, number of children vs. childhood health, sex vs. age, Sweden vs. number of children, childhood finances vs. number of children | Sex vs. childhood finances, number of children vs. childhood finances, living with spouse vs. childhood finances, number of children vs. fluency, number of children vs. childhood health, Estonia vs. number of children, sex vs. fluency, error term education vs. error term income | Age vs. childhood health, sex vs. childhood finances, fluency vs. number of children, living with spouse vs. childhood finances, error term quality of life vs. error term net income, error term education vs. error term quality of life, error term quality of life vs. error term later life health, error term education vs. error term net income, Italy vs. sex, Italy vs. fluency | Sex vs. childhood finances, number of children vs. childhood finances, fluency vs. number of children, sex vs. number of children, age vs. childhood health, France vs. age, The Netherlands vs. number of children, age vs. The Netherlands, error term quality of life vs. error term net income | Sex vs. childhood finances, number of children vs. Austria, sex vs. age, living with spouse vs. childhood finances, error term net income vs. error term quality of life, error term net income vs. error term education |
